# Rapid Phospholipid Turnover after Surfactant Nebulization in Severe COVID-19 Infection: A Randomized Clinical Trial

**DOI:** 10.1164/rccm.202110-2279LE

**Published:** 2021-12-07

**Authors:** Anthony D. Postle, Howard W. Clark, Jim Fink, Jens Madsen, Grielof Koster, Madhuriben Panchal, Ratko Djukanovic, David Brealey, Michael P. W. Grocott, Ahilanandan Dushianthan

**Affiliations:** ^1^ University of Southampton Southampton, United Kingdom; ^2^ University Hospital Southampton National Health System Foundation Trust Southampton, United Kingdom; ^3^ University College London Hospital London, United Kingdom; ^4^ University College London Hospital Biomedical Research Centre London, United Kingdom; ^5^ Aerogen Pharma Corporation San Mateo, California; ^6^ University College London London, United Kingdom; ^7^ University College Hospitals London London, United Kingdom

*To the Editor*:

The severe acute respiratory syndrome coronavirus 2 (SARS-CoV-2) virus targets the ACE-2 receptor on type II alveolar epithelial (ATII) cells. ATII cells synthesize and secrete lung surfactant, and surfactant deficiency consequent of ATII cell dysfunction may contribute to progression to acute respiratory distress syndrome (ARDS) in coronavirus disease (COVID-19), but this has yet to be confirmed (1). Several clinical trials of surfactant for COVID-19 ARDS are ongoing, but there is a critical need to optimize dose concentration, dose frequency and duration, and mode of delivery of therapeutic surfactant administration. In this pilot study, we evaluated the effectiveness of a prototype breath-synchronized vibrating mesh nebulizer to deliver exogenous surfactant to patients ventilated for severe COVID-19 infection (2, 3) and report here endogenous surfactant status, turnover, and half-life of administered surfactant. These results have not been previously reported in abstract form.

## Methods

Patients (*n* = 10) were recruited within 24 hours of endotracheal intubation into a randomized control trial of nebulized surfactant in patients with COVID-19 ARDS (Clinical Trials number: NCT04362059) and were randomized 3:2 for surfactant and control arms. Results were compared with historic healthy control data (4). Surfactant was nebulized by an investigational vibrating mesh breath-actuated aerosol system (Aerogen Pharma), controlled by a flow sensor in the inspiratory limb of the ventilation circuit (3). Six patients were randomly allocated to receive three doses of 1,080 mg of Alveofact, a bovine surfactant widely used in preterm neonates (5), at T = 0, 8, and 24 hours. Tracheal aspirates were taken at 0, 8, 16, 24, 48, and 72 hours. Phospholipids were analyzed by electrospray ionization mass spectrometry (6). Results are presented as medians and interquartile ranges, with statistical significance determined by the Mann-Whitney test.

## Results

**Figure 1.**
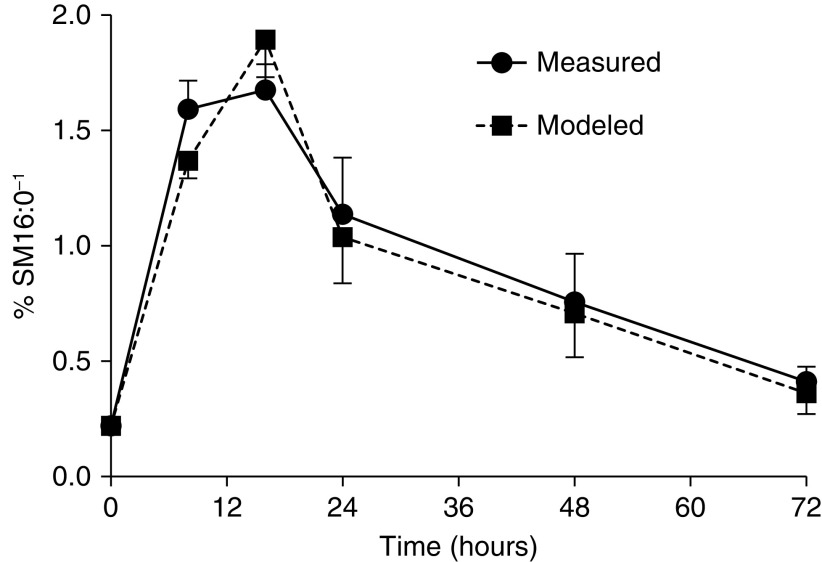
Alveofact turnover in tracheal aspirate samples. An index of exogenous surfactant was calculated as the reciprocal of the percentage concentration of palmitoylsphingomeyelin (SM16:0), the predominant sphingomyelin species that was taken as a marker of endogenous phospholipid. The solid circles represent the measured data, whereas the solid squares are the modeled data generated by fitting an iterative kinetic model of three sequential exponential decay curves (mean ± SEM).

Phospholipid analysis of baseline tracheal aspirates indicated that pulmonary surfactant was significantly compromised in patients ventilated for COVID-19 infection, with dipalmitoylphosphatidylcholine, the major surface-active component, being considerably reduced for all 10 patients (25.0, 20.9–29.4%) compared with healthy volunteers (46.8, 39.0–49.4% total phosphatidylcholine; *P* < 0.001) (data recalculated from reference [4]). Although phospholipid compositions were identical between COVID-19 control and surfactant groups at baseline (Table 1), initial concentrations of total phosphatidylcholine and phosphatidylglycerol, typically enriched in surfactant, were significantly lower than in Alveofact and healthy control groups (4) with corresponding increased concentrations of phospholipids characteristic of cell membranes such as phosphatidylserine and sphingomyelin (SM). The significant elevation of SM in tracheal aspirates from patients at T = 0 hours compared with Alveofact (10.5, 8.2–12.1, vs. 1.2, 1.0–1.3%; *P* < 0.01) provided a basis for calculation of exogenous surfactant turnover. Importantly for turnover calculations, sphingomyelin fractional concentration remained relatively constant over the study period in the control group (Table 1). By contrast, aspirate phospholipid composition after surfactant nebulization changed substantially to resemble that of Alveofact. Phosphatidylcholine concentration doubled from 39.8% to 79% at T = 8 hours, phosphatidylglycerol increased from 3.1 to 13.9% at T = 16 hours, and all other phospholipid classes substantially declined. These changes gradually reversed with time, such that by 72 hours, phosphatidylcholine concentration was 59.4%, phosphatidylserine was 9.1%, and SM was 5.3%, reflecting decreased concentration of exogenous surfactant. The wide variation of absolute aspirate phospholipid concentration owing to variable recovery precluded its use for turnover calculation. Instead, exogenous surfactant turnover was determined relative to that of endogenous lipid in tracheal aspirates, based on the assumption that concentration and composition of endogenous lipid did not change substantially over the 72-hour study period. Alveofact turnover was calculated from the reciprocal of the percentage concentration of palmitoylsphingomyelin (SM16:0) as a marker of endogenous lipid. This value was maximal for all patients between T = 8 and T = 16 hours and returned toward baseline values by T = 72 hours (Figure 1, solid circles). Kinetic models of these data were constructed by superimposing three first-order exponential decay curves at T = 0, T = 8, and T = 24 hours (Figure 1, solid squares), assuming equal amounts of Alveofact were delivered at each nebulization, with good agreement between measured and modeled values. The model indicated the median estimated half-life for Alveofact phospholipid was 7.6 hours (range, 1.8–20.8 h) in these six surfactant-treated patients.

**Table 1.** Concentration of Selected Phospholipid Classes in Tracheal Aspirate Samples over the 72-Hour Study Period for Control and Surfactant-treated Patients

	Phosphatidylcholine (*%*)	Phosphatidylglycerol (*%*)	Phosphatidylserine (*%*)	Sphingomyelin (*%*)
Alveofact composition (*n* = *4*)	80.4 (8.0–81.2)	10.8 (8.5–11.4)	0.8 (0.4–4.3)	1.2 (1.0–1.3)
Control group (*n* = *4*)				
0 h	48.0 (32.1–56.8)	2.2 (1.2–3.9)	22.6 (17.0–30.6)	9.8 (7.9–11.8)
8 h	48.5 (29.5–60.2)	4.2 (3.3–6.7)	18.0 (10.9–24.7)	9.1 (8.6–13.7)
16 h	38.0 (27.8–53.6)	2.7 (1.8–3.2)	17.3 (16.7–24.9)	9.0 (7.9–12.5)
24 h	34.1 (17.1–56.7)	3.5 (2.5–4.7)	20.5 (14.8–34.0)	10.4 (8.4–11.7)
48 h	38.7 (20.2–513)	3.3 (1.5–6.0)	27.0 (16.4–44.0)	10.9 (7.8–13.9)
72 h	35.4 (26.2–49.3)	2.1 (1.3–4.3)	32.4 (24.4–33.9)	11.8 (7.1–15.8)
Surfactant-treated group (*n* = *6*)				
0 h	39.8 (29.8–52.3)	3.1 (2.2–4.8)	23.5 (11.2–25.9)	10.9 (8.2–13.0)
8 h	79.0 (75.4–84.8)	11.7 (8.0–13.9)	1.2 (0.9–1.9)	2.0 (1.3–2.4)
16 h	76.4 (68.3–79.1)	13.9 (8.0–16.1)	1.1 (0.8–2.2)	1.9 (1.2–2.7)
24 h	75.9 (61.3–79.4)	11.9 (8.0–13.3)	1.5 (1.3–8.6)	3.0 (2.1–4.6)
48 h	67.8 (54.5–73.4)	10.1 (8.2–11.2)	7.0 (2.5–12.2)	3.7 (2.5–5.1)
72 h	59.4 (44.2–68.9)	9.8 (5.0–11.8)	9.7 (5.4–21.6)	5.3 (4.6–7.2)

Data are presented as median (interquartile range).

## Discussion

This is the first study to evaluate exogenous surfactant pharmacokinetics in patients with COVID-19; the rapid turnover of administered surfactant has direct implications for design of surfactant therapy trials in both patients with COVID-19 and those with other ARDS. It may help clarify why the transient improved oxygenation in most adult ARDS studies after surfactant supplementation was not sustained (7). Our findings support the hypothesis that pulmonary surfactant deficiency may play an important role in patients mechanically ventilated for severe COVID-19. This is the first detailed analysis of phospholipid compositional changes after administration of a therapeutic dose of exogenous surfactant to any patient group; it sampled tracheal aspirates instead of BAL to minimize health care–associated infections and prevent patient desaturation during frequent bronchoscopy. The novel methodology to estimate turnover based on the different lipid profiles of Alveofact and tracheal aspirates was analogous to the lecithin to sphingomyelin ratio (8) and rested on the assumption that endogenous phospholipid concentration was constant over 72 hours in the treatment group but was diluted by exogenous surfactant. Importantly, although the index of surfactant turnover was not a direct measure of exogenous surfactant concentration, as the upper value is set by the concentration of SM16:0 in Alveofact, it enabled iterative kinetic modeling to estimate half-life from three first-order exponential decay curves superimposed at T = 0, T = 8, and T = 24 hours. The resultant modeling of the Alveofact index was a reasonable fit to the measured data (Figure 1) and indicated a rapid turnover of exogenous surfactant in the airways and presumably the alveolus, such that little material remained at T = 72 hours. This data will be correlated with clinical variables in a future substantive report. This conclusion broadly agrees with the one other report of surfactant kinetics in patients with ARDS, which followed the loss of surfactant labeled with a tracer amount of ^13^C-palmitate in dipalmitoylphosphatidylcholine (9). This rapid turnover of exogenous surfactant may be one contributor to the failure of clinical trials of surfactant therapy in ARDS, in addition to the heterogeneity of the patient population and multiple initiating factors. Our results suggest that more individualized and prolonged surfactant administration may be required to give time for recovery of the lungs in severe COVID-19.
